# EVOLUTION OF GOAL SETTING AND ATTAINMENT OVER REPEATED CYCLES OF BOTULINUM TOXIN A FOR UPPER LIMB SPASTICITY IN REAL-LIFE CLINICAL PRACTICE: LONGITUDINAL ANALYSES FROM THE OBSERVATIONAL ULIS-III COHORT STUDY

**DOI:** 10.2340/jrm.v58.45139

**Published:** 2026-04-28

**Authors:** Lynne TURNER-STOKES, Klemens FHEODOROFF, Jorge JACINTO, Mathieu BENETEAU, Pascal MAISONOBE, Christian HANNES, Stephen ASHFORD

**Affiliations:** 1 Department of Palliative Care, Policy and Rehabilitation, Faculty of Nursing, Midwifery and Palliative Care, King’s College London, London; 2 Regional Hyper-acute Rehabilitation Unit, Northwick Park Hospital, London North West Healthcare NHS Trust, London, UK; 3 Neurorehabilitation, Gailtal-Klinik, Hermagor, Austria; 4 Centro de Medicina de Reabilitaçãode Alcoitão, Serviço de Reabilitação de adultos 3, Estoril, Portugal; 5 Department of Biometry, Ipsen, Paris, France; 6 Medical Affairs, Ipsen, Munich, Germany

**Keywords:** botulinum toxin A, goal attainment scaling, physical therapies, post-stroke spasticity, stroke rehabilitation

## Abstract

**Objective:**

This post-hoc analysis evaluated how goal attainment evolved over repeated cycles of botulinum toxin A (BoNT-A) treatment in adults with upper-limb spasticity.

**Methods:**

ULIS-III (NCT02454803) was a 2-year observational study involving adults treated with BoNT-A. The analysis included 538 patients who received ≥ 4 BoNT-A injection cycles and had Goal Attainment Scaling (GAS) assessments for each of the first 4 cycles. GAS T-scores were used to measure treatment response. Multivariate models assessed predictors of achieving a GAS T-score ≥ 50 and being in the top tertile (best) of responders.

**Results:**

Patients generally maintained consistent goal domains across cycles. Mean change in GAS T-scores remained above the minimal clinically important difference of 10 throughout. The proportion of patients achieving a GAS T-score ≥ 50 increased from 64.5% in Cycle 1 to 75.8% in Cycle 4. Use of injection guidance techniques significantly increased the odds of achieving treatment goals (OR 1.92; 95% CI 1.45–2.55) and being a “best” responder (OR 2.44; 95% CI 1.47–4.06). Higher BoNT-A doses were also associated with better outcomes.

**Conclusions:**

Repeated BoNT-A treatment supports sustained goal attainment in upper limb spasticity. Success rates improve with each cycle, particularly when guided injection techniques are used.

## LAY ABSTRACT

The ULIS-III study followed people with upper limb (arm and shoulder) spasticity who were treated with botulinum toxin A over 2 years in routine practice. The aim was to understand how well patients achieved their personal treatment goals across repeated treatment cycles. Researchers focused on 538 individuals who received at least 4 cycles of botulinum toxin A and used a method called Goal Attainment Scaling to track progress. This approach allowed patients to set goals that were meaningful to them, such as improving movement or reducing pain. The study found that most patients continued to set the same types of goals over repeated injections and while the improvement in Goal Attainment Scaling scores became slightly smaller, the likelihood of achieving goals increased with each cycle. This suggests a cumulative benefit from repeated treatment. Importantly, how the treatment was delivered had a bigger impact on success than patient characteristics like age or sex, indicating that most people have potential to benefit from this repeated treatment. Using techniques to guide the injection accurately into the target muscles – such as ultrasound, electromyogram, or electrical stimulation – was strongly linked to better outcomes. Higher doses of botulinum toxin A also improved the chances of success, although most patients were treated with doses below the maximum allowed. Overall, the study supports the long-term use of botulinum toxin A for managing upper limb spasticity and highlights the importance of setting clear goals and using precise injection techniques. These findings can help clinicians tailor treatment plans and improve outcomes for people living with spasticity.

Upper limb spasticity is a common, lifelong consequence of the upper motor neurone syndrome that is caused by stroke, traumatic brain injury, and other conditions associated with damage to the central nervous system. The efficacy and safety of botulinum toxin type A (BoNT-A) in the management of upper limb spasticity in adults has been well established in controlled clinical studies (1–3), and its repeated use over time to maintain the benefits of treatment is embedded in routine clinical practice (4–6). However, while it has been relatively easy to show improvements in spasticity (as measured by the modified Ashworth scale [MAS] or Tardieu scale) over repeated cycles, it has been harder to demonstrate treatment-related improvements in functional ability (7, 8). A key reason for this is the lack of a single standardized measure that is able to account for the diversity of clinical presentations as well as individual expectations and goals for spasticity management (9).

The Upper Limb International Spasticity (ULIS) study programme (10) was a series of international observational studies, conducted in the context of real-life clinical practice over 10 years, that consistently used Goal Attainment Scaling (GAS) as the primary person-centred outcome to evaluate the effectiveness of rehabilitation across diverse treatment goals (10–12). Starting in 2008, the original aim of the programme was to examine predictors of the best (and worst) response to treatment (10). We have previously reported the primary analysis of the ULIS-III study, which evaluated the effectiveness of repeated BoNT-A treatment at the *population level* recorded at each treatment cycle (13). In a published post-hoc population-level analysis, predictors of a positive response to treatment on GAS (i.e., achievement of the minimally important clinical difference of at least 10 points on the GAS T-score between baseline and follow-up for each cycle) were use of injection guidance techniques (*p* = 0.001), female sex (*p* = 0.031) and abobotulinumtoxinA therapy (vs onabotulinumtoxinA; *p* < 0.001) (14). However, these analyses were unable to capture the evolution of goal attainment over repeated cycles at the *patient level*.

We report here post-hoc analyses that explore in greater detail the longitudinal effects of repeated BoNT-A injection on GAS T-score at the patient level. We describe the evolution of goal attainment for the sub-group of patients who completed at least 4 treatment cycles to examine their outcomes over successive cycles. We also set out to evaluate the predictors of the “best” and “worst” responses, as defined by the upper and lower tertiles (i.e., > 67% and < 33%) of change in GAS T-scores.

## METHODS

### Study design and participants

Full details of the ULIS-III methodology have been described previously (13, 15). In brief, ULIS-III was an international observational, prospective, longitudinal cohort study following adult patients (≥ 18 years old) treated for upper limb spasticity over 2 years. Participants were treated at specialist centres with ≥ 1 cycle of botulinum toxin type A (BoNT-A, all commercially available formulations were permitted) in combination with an individualized physical management programme. The clinical decision to prescribe BoNT-A was taken before and independently of the investigators’ decision to offer study participation. All treatments (pharmacological and non-pharmacological) were given in accordance with local practice. Patients could be naïve to BoNT-A treatment or have had previous BoNT injections, provided there had been at least a 12-week interval between the last injection and study entry.

Each cycle included at least 1 goal-setting visit and the following assessment/goal review visit; the timing of follow-up was at the discretion of the treating investigator. Patients could set up to 3 goals (1 primary and 2 secondary) per cycle. Goal attainment was assessed using the *GAS – Evaluation of Outcome for Upper limb Spasticity* (GAS-eous) tool, in which goals were categorized into 6 goal areas: passive function, active function, pain, range of movement, involuntary movements, and mobility.

### Ethics

The study was conducted in compliance with Guidelines for Good Pharmacoepidemiology Practices (GPP) and all sites were located in countries with marketing authorization for use of BoNT-A in upper limb spasticity management. Ethical approval and written informed consent to the recording of anonymous data was obtained in countries where this was required. The study is registered with clinicaltrials.gov with the number NCT02454803.

### Statistical analysis

To assess the evolution of goal attainment over time at patient level, we selected the subgroup of patients who had received at least 4 injection cycles of BoNT-A with GAS T-scores available for each of these 4 cycles (hereafter referred to as the “4-cycle population”). As previously reported (13), the median number of treatment cycles completed within the 2-year study period was 4 (range 1–9) and the 4-cycle population included sufficient numbers of participants treated with each BoNT-A formulation for analysis.

The GAS T formula is designed such that if (primary and secondary) goals are achieved as expected, the mean GAS T-score will be 50 with a standard deviation of ±10 (16). Mean and 95% confidence intervals (95% CI) GAS T-scores at the end of each of the 4 injection cycles as well as changes in GAS T (end of cycle score – start of cycle score) were assessed descriptively. Responder analyses evaluated the percentage of patients who achieved (a) a GAS T-score of ≥ 50 and (b) their primary goal in each cycle.

The relationship between being a “GAS T responder” (achievement of GAS T-score of ≥ 50 per cycle) or a “best responder” (defined as the top tertile [33%] of patients showing the greatest improvement from Baseline) and any potential influential patient- or treat-ment-related characteristic was tested on the 4-cycle population using a multivariate mixed model for repeated measures (MMRM) analysis on the first 4 injection cycles. The first step included a univariate mixed model on a broad range of Baseline characteristics and treatment factors, chosen for their clinical relevance. Baseline characteristics included age; sex (female vs male); dominance of the affected limb (dominant vs non-dominant); lower limb spasticity at Baseline (yes vs no); time since onset of the event leading to ULS (as continuous data and categorical: ≤ 6 months, [6–12 months], [12–24 months], and > 24 months); and previous treatment with BoNT-A for ULS (yes vs no). Treatment factors included primary goal area at first injection cycle; mean GAS T-score at Baseline (start of each cycle); mean number of muscles injected across cycles; mean indexed dose across the first 4 injection cycles (units are standardized to percentage of maximum authorized dose for the relevant country across the first 4 injection cycles); ≥ 75% injections given using an injection guidance technique (yes vs no); and total time spent on self-rehabilitation across cycles (as continuous data and categorical: < 3 h, 3–10 h, 10–24.5 h, and > = 24.5 h). All potential factors with a *p*-value < 20% were retained for the second step; other factors were disregarded. In the second step, two-sided pairwise tests of association between the factors retained in the first step were performed to identify non-independent factors using the following tests: Fisher’s exact test (2 categorical factors with a maximum of 2 modalities for each factor), χ^2^ test (2 categorical factors of which 1 factor with more than 2 modalities), Kruskal–Wallis test (1 categorical factor and 1 continuous factor) or a Spearman test (2 continuous factors) depending on the nature of the factors assessed. In case of confirmed association (*p*-value < 0.0001%), the decision on which variables to retain for the final step was made through consensus by the clinical steering committee. Factors selected in the second step were then included in a multivariate stepwise MMRM analysis. Retained factors were: cycle effect (forced in the model), factors effect with a type 3 *p*-value < 5%, and “factor*cycle” interactions with a type 3 *p*-value < 10%.

All statistical evaluations were performed using the Statistical Analysis System (SAS V.9.4; SAS Institute Inc, Cary, NC, USA). Statistical tests were two-sided and performed at the 5% level of significance. In this observational study design, missing data were expected, and no imputations were made. Sample sizes are reported for each cycle.

## RESULTS

### Patient disposition and baseline characteristics

Just over half (538 of 1,004, 53.6%) enrolled participants underwent ≥ 4 BoNT-A injection cycles with GAS T-scores at each cycle and were included in the 4-cycle population. Baseline characteristics for the 538 patients included in the 4-cycle population were like those previously reported for the broader effectiveness population (13) and are provided in Table SI. Most patients in the 4-cycle population were living with chronic spasticity, mostly due to stroke (81.8%); the mean (95% CI) time since onset of spasticity was 8.0 (7.1, 8.8) years.

The most commonly injected BoNT‑A preparation at Cycle 1 was abobotulinumtoxinA (*n* = 337, median total dose 920 U), followed by onabotulinumtoxinA (*n* = 137, median total dose 210 U) and incobotulinumtoxinA (*n* = 62, median total dose 250 U). The mean (SD) number of muscles injected across cycles was 5.9 (2.4).

### Evolution of goal setting and attainment across the 4 treatment cycles

**Fig. 1:**
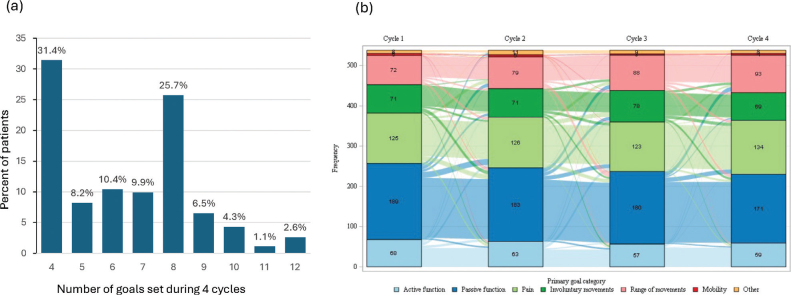
Goal setting across 4 cycles: (A) number of primary and secondary goals set; (B) Sankey diagram* showing changes in primary goal domains across the 4 treatment cycles. *Each pathway width is proportional to the number of participants following the pathway.

Patients had a median (Q1, Q3) of 6.5 (4, 8) primary and secondary goals set across the 4 treatment cycles (Fig. 1A). One in 3 patients had just 4 goals recorded (indicating that they only set primary goals) and about a quarter of patients had 8 goals recorded, usually reflecting the setting of 1 primary and 1 secondary goal per cycle. As shown in Fig. 1B, most patients retained the same primary goal area across the 4 cycles, with the most common changes being from passive function goals to other goals.

**Fig. 2:**
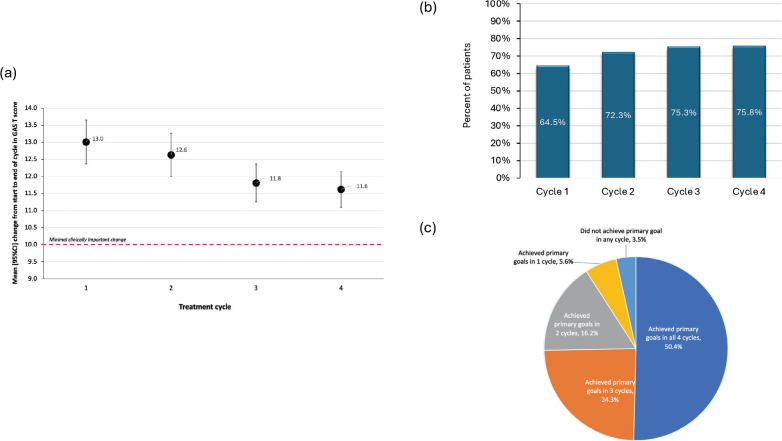
Goal attainment (4-Cycle Population). (A) mean [95%CL] change in GAS T-scores per cycle; (B) overall goal achievement per cycle; (C) primary goal achievement over 4 cycles.

Mean GAS T-scores at the end of each of the 4 cycles indicated goals were consistently achieved as expected, with scores as follows (mean [95% CI]: Cycle 1, 50.1 [49.5, 50.8]; Cycle 2, 49.9 [49.3, 50.5]; Cycle 3, 49.5 [49.0, 50.0]; and Cycle 4, 49.6 [49.0, 50.1]). Accordingly, improvements in GAS T-scores remained above the minimally clinically relevant change of 10 points in all 4 cycles (Fig. 2A). The percentage of patients who achieved a GAS T-score of ≥ 50 increased slightly over the successive cycles, from 64.5% in Cycle 1 to 75.8% in Cycle 4 (Fig. 2B). Looking at primary goal achievement only, half of patients (271/538, 50.4%) achieved their primary goals in all 4 cycles; 131/538 (24.3%) patients achieved goals in all but 1 cycle, 87/538 (16.2%) achieved their goals in half of the cycles, and 30/538 (5.6%) achieved their goals in just 1 cycle. The remaining 19 patients (3.5%) did not meet their primary goal in any cycle.

### Prognostic indicators for being a GAS T responder to treatment

Multivariate analyses confirmed that the odds of being a GAS T responder to treatment increased over consecutive cycles. For example, the chances of a patient responding to treatment were 1.76 (1.38,2.24) times higher in Cycle 4 than in Cycle 1 (Table I). In addition, patients who were injected under injection guidance in ≥ 3 of the 4 cycles were 1.92 (95% CI 1.45; 2.55) more likely to achieve a GAS T-score of ≥ 50 than those injected under guidance in ≤ 2 cycles. Patients who required the most therapy time (≥ 24.5 h per week) were significantly less likely to achieve their treatment goals than those who required < 3 h of therapy time per week (odds ratio of 0.49 [0.34; 0.71]).

**Table I:** Prognostic indicators for a positive response to treatment

Parameter	Variable	Odds ratios (95% CI)	*p*-value
Prognostic indicators for achieving a GAS T-score of ≥ 50				
Use of any injection guidance technique at ≥ 3 of 4 injection cycles	Yes vs no	1.92 (1.45; 2.55)	< 0.0001
Cycle effect	Overall		< 0.0001
	Cycle 2 vs Cycle 1	1.45 (1.16, 1.83)	0.0013
	Cycle 3 vs Cycle 1	1.71 (1.32, 2.20)	< 0.0001
	Cycle 4 vs Cycle 1	1.76 (1.38, 2.24)	< 0.0001
Total therapy time (h/week)	Overall		0.002
	3–10 h vs < 3 h	0.98 (0.66; 1.46)	0.9341
	10–24.5 h vs < 3 h	0.77 (0.52; 1.14)	0.1937
	≥ 24.5 h vs < 3 h	0.49 (0.34; 0.71)	0.0002
Prognostic indicators for “best” response (top 33% of responders)				
Use of any injection guidance technique at ≥ 3 of 4 injection cycles	Yes vs no	2.44 (1.47, 4.06)	0.0006
Mean index dose across injection cycles	Increase of 1 unit*	1.01 (1.00, 1.01)	0.002

* Units are standardized to mean index dose adjusted for formulation.

Use of injection guidance in ≥ 3 of the 4 cycles was also a significant predictor of being a “best responder” to treatment. Patients who were injected under injection guidance were 2.44 (95% CI 1.47; 4.06) more likely to be a best responder than those injected in ≤ 2 cycles. Higher BoNT-A doses also predicted the chances of “best” response. For each increase of 1 unit of mean index BoNT-A dose, the chances of being in the top tertile of response increased by 1%.

## DISCUSSION

This *post-hoc* analysis of the ULIS-III study evaluated the patient-level, longitudinal effects of repeated injections of BoNT-A as part of an integrated rehabilitation programme over 2 years. Results support the consistent effectiveness of treatment over repeated cycles and demonstrate that the odds of achieving treatment goals increase over consecutive cycles. The study found that how BoNT-A was delivered mattered more than patient characteristics (e.g., age or sex). Specifically, using an additional injection guidance technique (in addition to use of anatomical landmarks), such as electrical stimulation, electromyography, and ultrasound, to help place the injection accurately was strongly linked to better outcomes. Higher doses of BoNT-A were also a prognostic factor for being a “best” responder.

To our knowledge, this is the first paper to explore patient-level longitudinal outcomes for goal attainment scaling (GAS) as the primary measure, from repeated injections of BoNT-A for upper limb spasticity in a large real-life observational study of routine clinical practice. As in the primary “population-level” analysis (13), change in GAS T-scores (including primary and secondary goals) remained above the minimal clinically important change of 10 (17), confirming that patients treated for ≥ 4 cycles continue to benefit from repeat treatment cycles. The present “patient-level” analyses extend this finding by showing that patients tend to prioritize the same goal areas and that, despite a diminishing change in GAS T-score from baseline, the chances of achieving treatment goals increase over successive cycles. While this may to some extent reflect learning in goal setting, so that patients and their treating teams set goals that their previous experience has shown to be achievable, it may also reflect changes in the underlying presentation with continued treatment, as well as practice effects. This is supported by emerging findings from analysis of changes in standardized measures across successive cycles for the individual goal domains in the ULIS-III dataset. For example, recent analyses by separate goal domain have shown evidence of a cumulative effect of repeated treatment on pain (18) and passive function (19).

An overarching aim of the entire ULIS programme was to understand who the best responders to BoNT-A treatment are. Of note, no demographic factors significantly predicted goal achievement. Indeed, in contrast to previous population-level analyses (14), female sex was not found to be a significant predictor of response. Instead, we found that optimising technique by using injection guidance was the strongest predictor of long-term treatment success. Similar results have recently been reported for another longitudinal, observational study following patients treated with abobotulinumtoxinA for lower limb spasticity (20). Patients treated for lower limb spasticity using at least 1 guidance technique were almost twice as likely to achieve their treatment goal than those injected without a guidance technique (20). Although we were unable to analyse differences between the different techniques, a recent Bayesian network analysis of 6 studies has further proposed the following hierarchy of techniques when treating limb spasticity: ultrasound ≥ electrostimulation > electromyography > manual needle placement (21). A significant advantage for ultrasound is that it enables real-time visualization of needle placement when accuracy is required, such as in the localization of fascicles within a muscle or in targeting small, overlapping muscles. Conversely, both electrical stimulation and electromyography offer superior precision in identifying motor endplates, which can result in more effective blockade of neurotransmission.

Increasing BoNT-A dose was also found to be a significant predictor of being a “best” responder. However, this observation must be interpreted in the context of the relatively low doses used in the study. Indeed, median doses of BoNT-A were consistently lower than the maximum approved doses per formulation, suggesting that clinicians prefer to dose lower than allowed. Considering the good rates of goal achievement, this appears reasonable for most patients; however, in the case of underachievement of goals, our data suggest that dosing should be one of the first considerations. We do not suggest using higher than recommended doses in most patients. While total therapy time was found to be an overall negative predictor of treatment success, detailed analysis showed that the negative relationship was driven by the patients who required several hours of therapy per week (compared with < 3 h per week) with qualified physiotherapists, occupational therapists, or therapy assistants. This likely reflects the severity of their condition rather than the impact of the therapy intervention. In accordance with the observational nature of the study, patients could receive any type of therapy individualized according to their needs.

### Strengths and limitations

Key strengths of the ULIS-III study include its large sample size, broad international representation, and real-world design, all of which enhance the generalizability of the findings. This is further supported by the inclusion of all aetiologies and all BoNT-A products. Unlike prior population-based analyses of the ULIS study (13, 22), we set out to analyse the longitudinal effects of repeated BoNT-A injection at the patient level.

However, we acknowledge several limitations. The absence of a control group and the presence of missing data are inherent challenges in observational studies conducted in routine clinical settings. Analyses at the domain level do not capture *within*-domain differences in goal statements (e.g., setting of more ambitious goals over time), where qualitative analyses may be more informative. Most patients in this study were treated with abobotulinumtoxinA, which may have introduced selection bias, although several centres used all 3 available products. Indeed, while we originally stated our intent to examine longitudinal goal attainment over 4–6 cycles (13), we found that the 4-cycle population provided the most robust population for analysis across all 3 BoNT-A formulations. This cohort represented more than half of the original effectiveness population, and the observation that the median number of treatment cycles completed in the overall study population was 4 (range 1–9) (13) supports the view that analyses of the 4‑cycle population are broadly representative of the study population. We did not formally compare outcomes in this selected subsample with those of patients not included in the longitudinal analyses and therefore cannot exclude systematic differences between these groups. For example, participants who required 6 or 7 treatment cycles within a 2-year timeframe might well represent a more severe population with different treatment goals and needs. The study was not designed or powered to compare these products directly. Finally, although understanding the best responders was the initial goal of the whole ULIS programme, it must be understood that the analyses described were applied *post-hoc* and without adjusting for multiplicity.

### Conclusion and future research

Our data highlight the complexity of the rehabilitation process and the need to learn from prior treatment cycles to optimize long-term outcomes. The positive outcomes from ULIS-III underscore the critical role of precise and transparent goal setting and optimization of injection techniques during BoNT-A treatment.

## Supplementary Material


