# Supplementary material for: EVOLUTION OF GOAL SETTING AND ATTAINMENT OVER REPEATED CYCLES OF BOTULINUM TOXIN A FOR UPPER LIMB SPASTICITY IN REAL-LIFE CLINICAL PRACTICE: LONGITUDINAL ANALYSES FROM THE OBSERVATIONAL ULIS-III COHORT STUDY
Source: J Rehabil Med. 2026 Apr 28;58:45139. doi: 10.2340/jrm.v58.45139 (PMC13135245; doi:10.2340/jrm.v58.45139)
Supplement: Supplementary file 1 [file JRM-58-45139-s1.pdf]

Appendix

Supplementary Table 1. Baseline characteristics of the 4-Cycle population

**Parameter**	**Effectiveness Set (N=953)[1]**	**4-Cycle Population (N=538)***
Age (years); mean (SD)	54.0 (15.3)	52.2 (15.6)
Sex; n (%) Male	537 (56.3%)	300 (55.8)
Time since onset of the event leading to upper limb spasticity (years)		
Mean (SD)	7.6 (9.4)	8.0 (9.8)
Median [IQR]	4.2 [8.1]	4.7 [8.9]
Diagnosis of condition leading to upper limb spasticity; n (%)		
Acquired brain injury (stroke/trauma/other)	870 (91.3%)	494 (91.8%)
Spinal cord injury	15 (1.6%)	6 (1.1%)
Progressive neurological condition	20 (2.1%)	8 (1.5%)
Congenital	44 (4.6%)	28 (5.2%)
Other	4 (0.4%)	2 (0.4%)
Aetiology; n (%)		
Trauma	71 (7.5%)	47 (8.7%)
Vascular (infarct or haemorrhage)	786 (82.5%)	440 (81.8%)
Hypoxic	25 (2.6%)	16 (3.0%)
Inflammatory/infective	15 (1.6%)	11 (2.0%)
Tumour	19 (2.0%)	9 (1.7%)
Degenerative	12 (1.3%)	2 (0.4%)
Cerebral palsy	10 (1.0%)	6 (1.1%)
Other	15 (1.6%)	7 (1.3%)
Spasticity distribution; n (%)		
Focal (part of the limb)	190 (19.9%)	100 (18.6%)
Regional	763 (80.1%)	438 (81.4%)
Affected limb; n (%)		
Right arm	409 (42.9%)	236 (43.9%)
Left arm	488 (51.2%)	266 (49.4%)
Both arms	56 (5.9%)	36 (6.7%)

**Not all patients had assessments of impairments; data are presented for patients with available data.*

1. Turner-Stokes L, Jacinto J, Fheodoroff K, Brashear A, Maisonobe P, Lysandropoulos A, et al. Longitudinal goal attainment with integrated upper limb spasticity management including repeat injections of botulinum toxin A: Findings from the prospective, observational Upper Limb International Spasticity (ULIS-III) cohort study. J Rehabil Med 2021;53(2):jrm00157.
